# Bbvac: A Live Vaccine Candidate That Provides Long-Lasting Anamnestic and Th17-Mediated Immunity against the Three Classical *Bordetella* spp.

**DOI:** 10.1128/msphere.00892-21

**Published:** 2022-02-23

**Authors:** Monica C. Gestal, Laura K. Howard, Kalyan K. Dewan, Eric T. Harvill

**Affiliations:** a Department of Infectious Diseases, College of Veterinary Medicine, University of Georgia, Athens, Georgia, USA; Baylor College of Medicine

**Keywords:** *Bordetella bronchiseptica*, *Bordetella pertussis*, *Bordetella parapertussis*, vaccine, mucosal immunity, antibodies, *Bordetella*, immunity, mucosal vaccines

## Abstract

Acute pathogens such as Bordetella pertussis can cause severe disease but are ultimately cleared by the immune response. This has led to the accepted paradigm that convalescent immunity is optimal and therefore broadly accepted as the “gold standard” against which vaccine candidates should be compared. However, successful pathogens like B. pertussis have evolved multiple mechanisms for suppressing and evading host immunity, raising the possibility that disruption of these mechanisms could result in substantially stronger or better immunity. Current acellular B. pertussis vaccines, delivered in a 5-dose regimen, induce only short-term immunity against disease and even less against colonization and transmission. Importantly, they provide modest protection against other *Bordetella* species that cause substantial human disease. A universal vaccine that protects against the three classical *Bordetella* spp. could decrease the burden of whooping cough-like disease in humans and other animals. Our recent work demonstrated that *Bordetella* spp. suppress host inflammatory responses and that disrupting the regulation of immunosuppressive mechanisms can allow the host to generate substantially stronger sterilizing immunity against the three classical *Bordetella* spp. Here, we identify immune parameters impacted by *Bordetella* species immunomodulation, including the generation of robust Th17 and B cell memory responses. Disrupting immunomodulation augmented the immune response, providing strong protection against the prototypes of all three classical *Bordetella* spp. as well as recent clinical isolates. Importantly, the protection in mice lasted for at least 15 months and was associated with recruitment of high numbers of B and T cells in the lungs as well as enhanced Th17 mucosal responses and persistently high titers of antibodies. These findings demonstrate that disrupting bacterial immunomodulatory pathways can generate much stronger and more protective immune responses to infection, with important implications for the development of better vaccines.

**IMPORTANCE** Infectious diseases are a major cause of morbidity and mortality in the United States, accounting for over 40 million hospitalizations since 1998. Therefore, novel vaccine strategies are imperative, which can be improved with a better understanding of the mechanisms that bacteria utilize to suppress host immunity, a key mechanism for establishing colonization. *Bordetella* spp., the causative agents of whooping cough, suppress host immunity, which allows for persistent colonization. We discovered a regulator of a bacterial immunosuppressive pathway, which, when mutated in *Bordetella* spp., allows for rapid clearance of infection and subsequent generation of protective immunity for at least 15 months. After infection with the mutant strain, mice exhibited sterilizing immunity against the three classical *Bordetella* spp., suggesting that the immune response can be both stronger and cross-protective. This work presents a strategy for vaccine development that can be applied to other immunomodulatory pathogens.

## INTRODUCTION

The classical *Bordetella* spp. are respiratory pathogens that can cause disease in humans (Bordetella pertussis, B. parapertussis) or a broad variety of mammals, including humans (B. bronchiseptica) (1–3). They are the causative agents of whooping cough or whooping cough-like disease, and, despite the international vaccination efforts to eradicate this disease, incidence has increased since the move to acellular vaccines (4–6). Current acellular vaccines are very safe and efficient in preventing severe symptoms of disease but are not able to prevent colonization of the nasal cavity and subsequent transmission to new hosts (7, 8). In addition, the immunity they produce wanes relatively rapidly (9–11), and there is growing evidence of vaccine-driven evolution of key vaccine antigens, leading to fears of even larger outbreaks (12–14). In 2014, the number of cases of whooping cough was estimated to be over 24 million worldwide (15), underscoring the imperative need for new and improved vaccines. Recent novel vaccine strategies have included new antigens and adjuvants (16), the use of live vaccines (17–21), and the use of outer membrane vesicles (22–24, 53). These efforts are focused on B. pertussis, although this is not the only *Bordetella* sp. that causes disease in humans. In the particular case of B. bronchiseptica, this is not only a problem in dogs that need to be vaccinated every 6 to 12 months (25–30, 54), but also in other animals, including pigs (31–37), causing increased morbidity in farm animals. Moreover, zoonoses from these animals have been also reported, indicating that there is a need for a universal vaccine. Fortunately, it has been shown that B. bronchiseptica can confer protection against another *Bordetella* spp. (38), providing an alternative for the development of novel vaccines against whooping cough-like disease.

In our previous work, we investigated the molecular mechanisms that *Bordetella* spp. utilize to respond to host inflammatory signals. We identified a sigma factor named *btrS* that increases expression of virulence factors in the presence of blood/serum (1), indicating that it might be involved in the response to host inflammation. To investigate the role of this sigma factor in modulating host immunity, we challenged mice with the parent strain, RB50, or an isogenic mutant lacking *btrS* (Bbvac). Our results revealed that Bbvac is cleared more rapidly from the respiratory tract and triggers faster and more robust Th17 immunity (3). When complemented with a plasmid-bearing intact *btrS*, persistent lung infection was largely restored.

Here, we characterize this mutant with disrupted immunomodulation (Bbvac) as a potential live vaccine, demonstrating that it induces sterilizing immunity against all three classical *Bordetella* spp., something the existing vaccines cannot do (39). Moreover, B. bronchiseptica is genetically stable and does not cause disease in immunocompetent humans, providing a high level of safety. In mice, Bbvac induced robust long-lasting protection for at least 15 months, which involved a combination of antibody and cellular Th17 responses that protected against both laboratory strains and recent clinical isolates of all three classical *Bordetella* spp.

## RESULTS

### Bbvac provides more robust protective immunity than classical *Bordetella* spp. vaccines or convalescent immunity.

**FIG 1:**
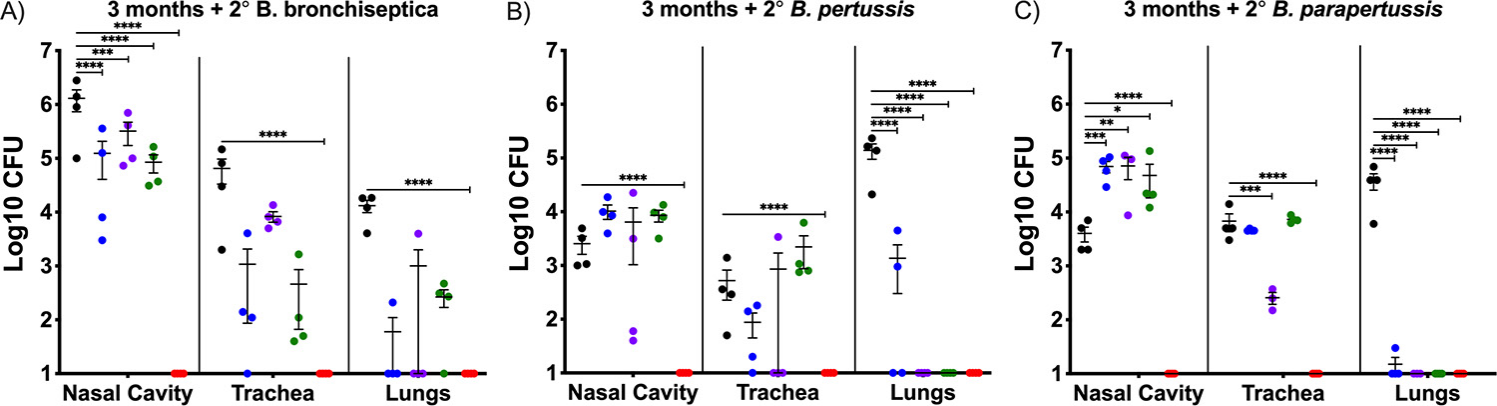
Bbvac confers sterilizing immunity against infection with the 3 classical *Bordetella* spp. C57BL/6J mice were intranasally inoculated with PBS (black) or PBS containing 5 × 10^5^ CFU of B. bronchiseptica (blue), Nobivac (green), or Bbvac (red). Another group was intraperitoneally vaccinated with 1/5 of Adacel (purple). Ninety days later, mice were intranasally challenged with PBS containing 5 × 10^5^ CFU of B. bronchiseptica (A), B. pertussis (B), or B. parapertussis (C). Mice were euthanized 7 days postinoculation, and colonies were enumerated in the respiratory tract. Two-way ANOVA, Dunnett's multiple-comparison test. *, *P* < 0.05; **, *P* < 0.01; ***, *P* < 0.001; ****, *P* < 0.0001. Error bars represent standard error of the mean. *n* = 4 per condition.

Our previous data revealed that a B. bronchiseptica mutant lacking *btrS* (RB50Δ*btrS*), here Bbvac, fails to suppress host immune responses, resulting in anamnestic protective immunity against reinfection with either B. pertussis, B. bronchiseptica, or B. parapertussis (3). To evaluate if the protection conferred by Bbvac is greater than the gold standard, which is the protection provided by convalescent immunity, groups of mice were challenged with wild-type B. pertussis (Fig. S1 in the supplemental material), B. bronchiseptica, or Bbvac and allowed to clear infection (antibiotics were provided after 28 days to clear B. bronchiseptica) to induce anamnestic immunity. Current commercially available vaccines were also included in this experiment, and additional groups of mice were vaccinated with the acellular vaccine Adacel (purple) or the veterinary live attenuated vaccine Nobivac (green). Mice sham vaccinated with phosphate-buffered saline (PBS; black) served as controls. Ninety days after vaccination/first challenge, mice were intranasally inoculated with wild-type B. bronchiseptica (Fig. 1A), B. pertussis (Fig. 1B), or B. parapertussis (Fig. 1C) with the standard inoculum (5 × 10^5^ CFU in 50 μL PBS) (3). On day 7 postinoculation (coinciding with the peak of infection) (40), mice were euthanized to enumerate bacterial burden in the nasal cavity, trachea, and lungs.

10.1128/msphere.00892-21.1 FIG S1 Bbvac confers better protection against B. pertussis than convalescence immunity. C57BL/6J mice were intranasally inoculated with PBS (black) or PBS containing 5 × 10^5^ CFU of B. bronchiseptica (blue), B. pertussis (purple), or Bbvac (red). The other group was intraperitoneally vaccinated with 1/5 of Adacel (green) and boosted 14 days later. Ninety days postinoculation/vaccination, mice were intranasally challenged with PBS containing 5 × 10^5^ CFU of B. pertussis. Mice were euthanized 7 days postinoculation, and colonies were enumerated in the respiratory tract. *n* = 4 per condition. Download FIG S1, TIF file, 1.9 MB. Copyright © 2022 Gestal et al. 2022 Gestal et al. https://creativecommons.org/licenses/by/4.0/ This content is distributed under the terms of the Creative Commons Attribution 4.0 International license.

The PBS sham vaccinated group (black) presented high numbers of bacteria with small to moderate variability in lungs, trachea, and nasal cavity following challenge with B. bronchiseptica (Fig. 1A), B. pertussis (Fig. 1B), and B. parapertussis (Fig. 1C). Vaccination with the current acellular vaccine Adacel (purple) robustly protected the lower respiratory tract from all three classical *Bordetella* spp., with one outlier discussed below. But Adacel was less protective against colonization of trachea or nasal cavity, as previously reported for mice (3), baboons (41, 42), and humans (7, 43). The veterinary vaccine, Nobivac (green), conferred only limited protection against B. bronchiseptica in nose, and variably in trachea and lungs, but did not completely clear the infection. Interestingly, Nobivac fully protected the lungs against infection with B. pertussis or B. parapertussis but had no detected effect on their numbers in trachea or nose. These data indicate that vaccination with commercially available vaccines, either Adacel or Nobivac, reduced numbers of bacteria in the lungs but did not protect the upper respiratory tract, which likely allows the bacteria to continue to be shed and transmitted. These results are consistent with observations that current vaccines can prevent severe forms of disease but do not prevent colonization, shedding, and transmission.

Examining convalescent immunity, the current gold standard of protective immunity, previous infection with B. bronchiseptica (blue) protected the lung against reinfection with B. bronchiseptica, B. pertussis, or B. parapertussis. However, protection was less strong in the trachea and was nominal or absent in the nasal cavity, indicating that convalescent immunity protects the lower respiratory tract but not middle and upper. Previous infection with B. pertussis (Fig. S1) conferred limited protection against B. pertussis and B. parapertussis in all three organs, allowing for colonization in middle and upper respiratory tracts. These data, similar to previously published results (44), indicate that convalescent immunity following infection with B. bronchiseptica or B. pertussis confers some level of protection against disease, especially in lower and middle respiratory tracts, but still allows for colonization of the nasal cavity.

Importantly, Bbvac (red) conferred sterilizing immunity against all three classical *Bordetella* spp., with none of the 10^5^ CFU inoculated bacteria detected in the respiratory tract, thereby outperforming not only the currently available vaccines but also convalescent immunity induced by either B. bronchiseptica or B. pertussis. These results belie the concept of convalescent immunity as the gold standard of best achievable protection against which vaccines should be measured and reveal that much better protective immunity can be achieved.

### Bbvac induces sterilizing immunity against diverse clinical isolates of B. bronchiseptica.

**FIG 2:**
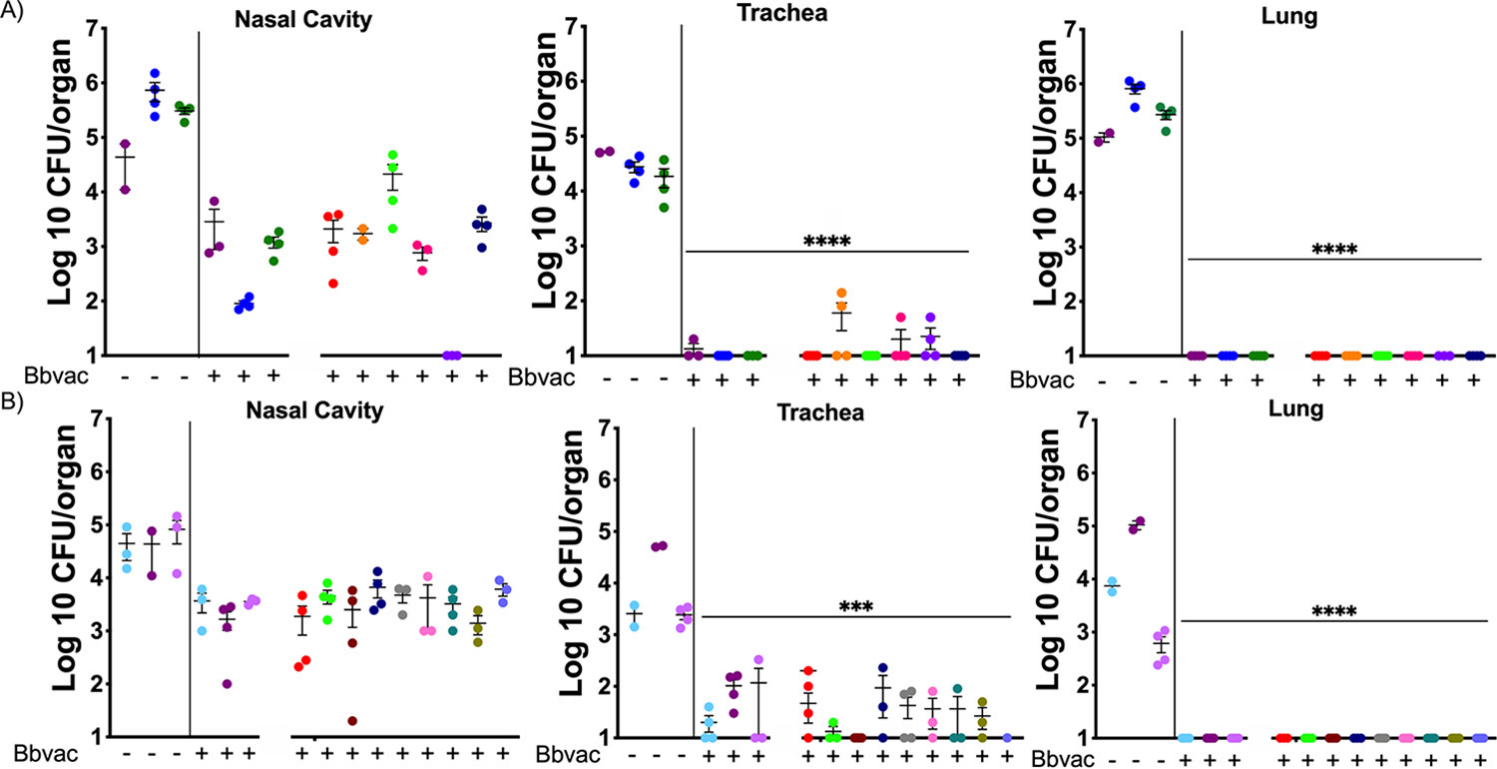
Bbvac confers robust protection against clinical isolates of B. bronchiseptica. C57BL/6J mice were intranasally vaccinated with PBS or PBS containing 5 × 10^5^ CFU of Bbvac. Three (A) and 7 (B) months after vaccination, mice were intranasally challenged with PBS containing 5 × 10^5^ CFU of different clinical isolates of B. bronchiseptica. Mice were euthanized 7 days postinoculation, and colonies were enumerated in the respiratory tract. Two-way ANOVA, Dunnett's multiple-comparison test. ***, *P* < 0.001; ****, *P* < 0.0001. Error bars represent standard error of the mean. *n* = 4 per strain and condition.

The evidence that Bbvac confers better protection against reinfection than convalescent immunity induced by prior infection with wild-type B. bronchiseptica or B. pertussis suggests potentially important clinical application as a live vaccine. To evaluate efficacy against clinical isolates, mice were intranasally vaccinated with Bbvac 3 and 7 months prior to challenge with 17 different clinical isolates of B. bronchiseptica (Fig. 2; Fig. S2; Table S2) (3, 45). Mice were euthanized at day 7 postchallenge, and bacterial numbers were enumerated in the respiratory tract.

10.1128/msphere.00892-21.2 FIG S2 Bbvac confers robust protection against clinical isolates of B. bronchiseptica. C57BL/6J mice were vaccinated with PBS (black) or PBS containing 5 × 10^5^ CFU of Bbvac (red). Ninety days later, mice were intranasally challenged with PBS containing 5 × 10^5^ of a clinical isolate of B. bronchiseptica (a total of 12 different clinical isolates were tested). Mice were euthanized at day 7 postinfection, and colonies were enumerated in the respiratory tract. Two-way ANOVA was performed. **, *P* < 0.001; ****, *P* < 0.0001. *n* = 4 mice per strain and condition. Download FIG S2, TIF file, 0.4 MB. Copyright © 2022 Gestal et al. 2022 Gestal et al. https://creativecommons.org/licenses/by/4.0/ This content is distributed under the terms of the Creative Commons Attribution 4.0 International license.

10.1128/msphere.00892-21.5 TABLE S2 Clinical isolates included in this study. List of clinical *Bordetella* spp. strains utilized to determine protection levels conferred by Bbvac. This table indicates if the strains were used to test protection at 3 months, 7 months, or both. Download Table S2, DOCX file, 0.02 MB. Copyright © 2022 Gestal et al. 2022 Gestal et al. https://creativecommons.org/licenses/by/4.0/ This content is distributed under the terms of the Creative Commons Attribution 4.0 International license.

Mice vaccinated with Bbvac 3 months earlier completely cleared all challenged clinical isolates from the lungs and had only very low levels of colonization in the trachea for one strain (Fig. 2A). Bacteria were detected in the nasal cavity, but the numbers were, on average, >99% reduced and, in all cases, >90% lower than the nonvaccinated group, greatly reducing the likelihood of bacterial shedding and further disease transmission. Importantly, disease is associated with bacteria in the trachea and lungs, where Bbvac reduced numbers of all strains by >99.9%, eliminating all, or nearly all, of this large inoculum within 7 days.

To determine long-term protection, we infected mice with the same clinical strains 7 months after Bbvac vaccination. Seven days after conventional challenge (50 μL of PBS containing 5 × 10^5^ CFU), less than 1% of the inoculated dose of any B. bronchiseptica strains remained (Fig. 2B). The lungs were completely protected, and the trachea was largely protected from all strains. Bbvac reduced numbers of all strains of B. bronchiseptica in the nose to a few thousand, a 99% reduction from the initial inoculum. These data indicate that even after 7 months post-Bbvac vaccination, there is complete protection of the lower respiratory tract and substantial protection in all respiratory organs. Overall, these data indicate that Bbvac confers lasting protection against diverse laboratory and clinical strains.

### Bbvac provides long-lasting immunity against classical *Bordetella* spp.

**FIG 3:**
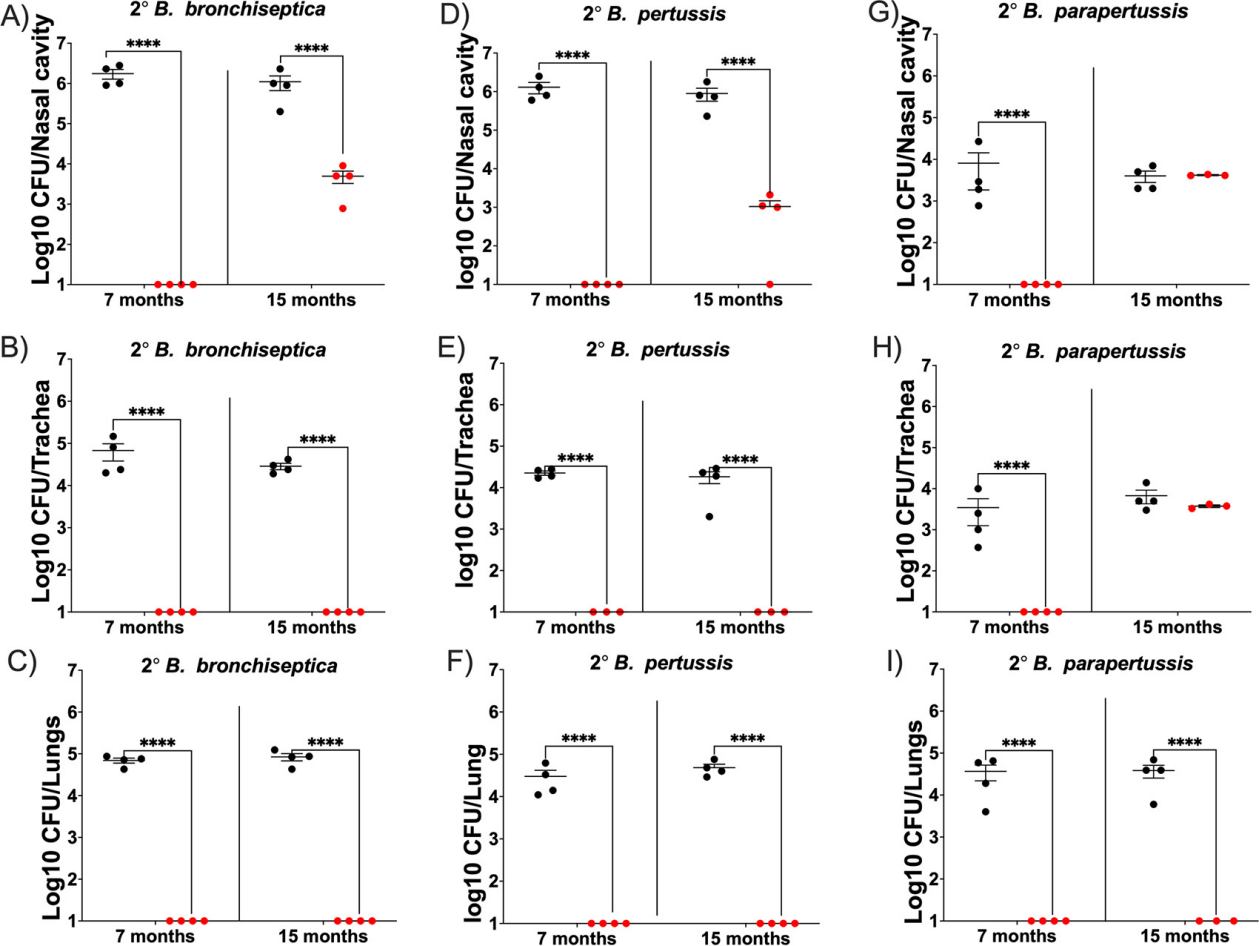
Bbvac confers robust protection for at least 15 months postvaccination. C57BL/6J mice were intranasally vaccinated with PBS (black) or PBS containing 5 × 10^5^ CFU of Bbvac (red). Seven and 15 months after vaccination, mice were intranasally challenged with PBS containing 5 × 10^5^ CFU of B. bronchiseptica (A, B, and C), B. pertussis (D, E, and F), or B. parapertussis (G, H, and I). Mice were euthanized 7 days postinoculation, and colonies were enumerated in the respiratory tract. Two-way ANOVA, Dunnett's multiple-comparison test. ***, *P* < 0.001; ****, *P* < 0.0001. Error bars represent standard error of the mean. *n* = 4 per strain and condition.

Our data demonstrating high levels of protection at even 7 months postvaccination are very promising, as one of the greatest concerns with the current acellular vaccines is that the immunity induced wanes quite rapidly (7) and that this is leading to an increase in the numbers of young adults that are suffering from whooping cough. Estimations of Tdap effectiveness within 2 years of vaccination revealed a 57.6% to 74.4% protection, indicating a decrease in effectiveness and limited duration of immunity (46). To investigate the length of protection, mice were vaccinated with Bbvac, and 3, 7, and 15 months postvaccination, we challenged them with B. bronchiseptica (Fig. 3A, B, and C), B. pertussis (Fig. 3D, E, and F), or B. parapertussis (Fig. 3G, H, and I). At 7 days postinoculation, mice were euthanized, and bacteria in respiratory organs were enumerated.

The sham vaccinated mice (black) had high levels of colonization in nasal cavity trachea and lungs following infection with the 3 classical *Bordetella* spp. Vaccination with Bbvac conferred sterilizing immunity of the nasal cavity against B. bronchiseptica (Fig. 3A), B. pertussis (Fig. 3D), and B. parapertussis (Fig. 3G) for at least 7 months postvaccination. Moreover, at 15 months postvaccination, Bbvac provided sterilizing immunity against B. bronchiseptica and B. pertussis in trachea and lungs and reduced numbers in the nasal cavity by >99%. It was similarly effective against B. parapertussis in the lungs but, interestingly, had no impact on the relatively low B. parapertussis numbers in the nasal cavity or trachea.

Excitingly, Bbvac vaccination provided at least 15 months of complete protection against B. bronchiseptica (Fig. 3C), B. pertussis (Fig. 3H), and B. parapertussis (Fig. 3I) in the lungs, where nearly all pathology is concentrated in this model. Even after 15 months (most of the life span of mice), there was only partial loss of protection against B. bronchiseptica and B. pertussis in the nose, but complete loss of protection against B. pertussis in both trachea and nose. Overall, these results demonstrate that Bbvac confers profound, protective immunity against lower respiratory tract disease caused by all three classical species. Even at 15 months postvaccination, there is near-complete protection in the lower respiratory tract, better than that conferred by commercial vaccines 3 months postvaccination or even that conferred by prior infection (Fig. 1).

### Bbvac promotes the generation of high-titer and long-lasting antibodies.

**FIG 4:**
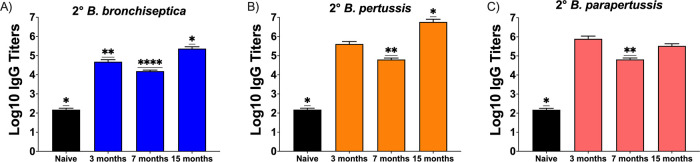
Bbvac promotes α-bordetellae antibodies for at least 15 months. C57BL/6J mice were intranasally vaccinated with PBS (black) or PBS containing 5 × 10^5^ CFU of Bbvac (colors). Three, 7, and 15 months after vaccination, mice were intranasally challenged with PBS containing 5 × 10^5^ CFU of B. bronchiseptica (A), B. pertussis (B), or B. parapertussis (C). Mice were euthanized 7 days postinoculation, and sera were collected for analysis. Antibody titers were determined to be reciprocal of the lowest dilution in which an OD of 0.1 was obtained. Mann-Whitney test was performed. ****, *P* < 0.0001. Error bars represent standard error of the mean. *n* = 4 per strain and condition.

To evaluate how protection in the long-term experiments above correlates to antibody titers, we compared serum collected from the above Bbvac vaccinated mice at 3, 7, and 15 months postvaccination. We did not evaluate antibody titers for currently available vaccines, as this information is well established in the literature (8, 47, 48), and our previous data (Fig. 1) showed that protection is not as robust as that conferred by Bbvac. To evaluate circulating total IgG levels, serum was collected at day 7 (coinciding with the peak of infection) from the 3-, 7- or 15-month vaccinated mice following intranasal challenge with B. bronchiseptica (Fig. 4A), B. pertussis (Fig. 4B), or B. parapertussis (Fig. 4C).

As expected, the naive uninfected control mice (Black) had low titers (∼100) of antibodies against the three *Bordetella* species, B. bronchiseptica (Fig. 4A), B. pertussis (Fig. 4B), and B. parapertussis (Fig. 4C). The Bbvac-vaccinated group presented very high titer (>100,000) of antibodies against B. bronchiseptica (Fig. 4A), B. pertussis (Fig. 4B), and B. parapertussis (Fig. 4C) at 3, 7, and 15 months postvaccination, indicating that Bbvac induces the generation of IgG antibodies that recognize all three classical *Bordetella* species and that these titers persist, or can be rapidly recalled, for at least 15 months.

To investigate if the antibody profile generated by Bbvac was different than that conferred by convalescent immunity or vaccination with the currently available vaccines, we performed Western blot analysis using sera from convalescent/vaccinated mice as primary antibody and whole-cell extract of B. bronchiseptica, B. pertussis, and B. parapertussis as antigens (Fig. S3). Bbvac serum antibodies recognized a broader and more diverse set of antigens than those generated by convalescent immunity or vaccination with Adacel or Nobivac, suggesting that BtrS suppresses the generation of antibodies to diverse antigens. Overall, these results demonstrate that vaccination with Bbvac induces the production of long-lasting IgG (Fig. 4) and more diverse (Fig. S3) systemic anti-*Bordetella* IgG antibodies, suggesting that humoral immunity might contribute to the robust protection conferred by Bbvac.

10.1128/msphere.00892-21.3 FIG S3 Bbvac generates more diverse a-*Bordetellae* antibodies. C57BL/6J mice were vaccinated with different commercially available vaccines and challenged with B. bronchiseptica or B. pertussis, vaccinated with Bbvac, or unvaccinated. Three months after vaccination, serum was collected and used as primary antibody for Western blot analysis. All the sections of the membrane were incubated together with the secondary antibody to promote binding competition (for each figure separately). We developed each panel to compare intensity. The results show the IgG antibody profile generated in mice against B. bronchiseptica (A) and B. pertussis (B). *n* = 3. Download FIG S3, TIF file, 1.9 MB. Copyright © 2022 Gestal et al. 2022 Gestal et al. https://creativecommons.org/licenses/by/4.0/ This content is distributed under the terms of the Creative Commons Attribution 4.0 International license.

### Bbvac-induced antibodies confer protection against challenge with B. pertussis and B. bronchiseptica.

**FIG 5:**
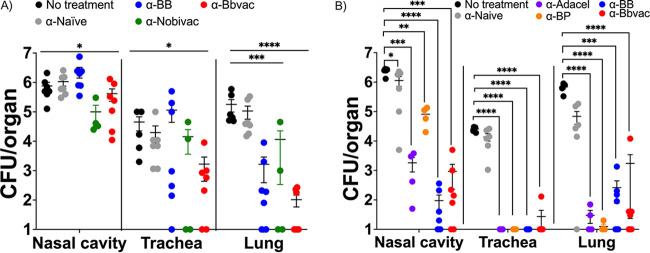
Bbvac-induced antibodies confer some protection against infection with B. bronchiseptica and B. pertussis. Serum from mice that were naive (α-naïve), inoculated with B. bronchiseptica (α-BB), inoculated with B. pertussis (α-BP), vaccinated with Nobivac (α-Nobivac), vaccinated with Adacel (α-Adacel), or vaccinated with Bbvac (α-Bbvac) was transferred to naive C57BL/6J mice. Three hours later, mice were intranasally challenged with B. bronchiseptica (A) or B. pertussis (B), and mice were euthanized 7 days postinoculation to enumerate colonies in the respiratory tract. Two-way ANOVA, Dunnett's multiple-comparison test. *, *P* < 0.05; **, *P* < 0.01; ***, *P* < 0.001; ****, *P* < 0.0001. Error bars represent standard error of the mean. *n* = 4 to 9 per strain and condition.

Our results show that Bbvac provides broad, robust, and more persistent protection than that provided by currently available vaccines or by wild-type B. bronchiseptica-induced convalescent immunity. To examine the relative contributions of circulating antibodies to protection, mice were vaccinated with Bbvac (red), B. bronchiseptica (blue), B. pertussis (orange), Nobivac (green), or Adacel (purple). Three months postvaccination, serum was collected. One hundred fifty microliters of serum was then adoptively transferred to naive mice intraperitoneally 3 h before inoculation with 50 μL of PBS containing 5 × 10^5^ CFU of B. bronchiseptica. (Fig. 5A) or B. pertussis (Fig. 5B). Mice were euthanized at 7 days postinoculation to enumerate bacteria in the respiratory tract.

As expected, the untreated mice (black), in which no serum was transferred, had high numbers (∼10^6^ CFU) of bacteria in the respiratory tract following inoculation with B. bronchiseptica (Fig. 5A) or B. pertussis (Fig. 5B). Similarly, mice that received serum from naive mice (gray) also had high numbers of B. bronchiseptica (Fig. 5A) and B. pertussis (Fig. 5B). Serum from Nobivac-vaccinated mice (green) appeared to reduce numbers of B. bronchiseptica in the lungs and trachea marginally, which was found to not be statistically significant (Fig. 5A). Serum from mice vaccinated with the current acellular vaccine, Adacel (purple), reduced numbers of B. pertussis by 95 to 100% in all three organs (Fig. 5B), being especially protective in the trachea but least protective in the nose. These results are consistent with prior studies showing that these vaccines induce antibodies that protect against lower respiratory infection/disease but are less protective against colonization of the upper respiratory tract (3, 43, 49).

Antibodies transferred from convalescent mice previously infected with B. bronchiseptica (blue) reduced B. bronchiseptica numbers in the lungs (Fig. 5A) but had no effect on B. bronchiseptica numbers in the nose or trachea. Interestingly, compared to B. pertussis convalescent-phase serum, B. bronchiseptica convalescent-phase serum provides similar protection against B. pertussis along the entire respiratory tract (Fig. 5B), supporting the previously observed cross-protection (38) that warrants further investigation. Moreover, B. pertussis convalescent-phase serum (orange) also conferred protection of the lower respiratory tract but was less effective than B. bronchiseptica convalescent-phase serum in reducing B. pertussis numbers in the nose. The observation that B. pertussis-induced serum was less effective against B. pertussis than B. bronchiseptica-induced serum is intriguing and consistent with proposed and observed immunomodulation of the host response, as discussed below.

Importantly, serum transferred from Bbvac-vaccinated mice (red) provided the best protection to the lungs against B. bronchiseptica (Fig. 5A); however, the transferred sera provided little protection against B. bronchiseptica in the trachea or the nasal cavity. Serum from Bbvac-vaccinated mice also reduced B. pertussis numbers by over 99.9%, which is as well as or better than any other sera (Fig. 5B). Overall, this indicates that anti-Bbvac antibodies confer protection against challenge with B. pertussis; however, it is noteworthy that serum antibodies alone did not confer the complete protection observed in Bbvac-immune animals, implying that other immune functions are involved.

### Prior Bbvac exposure elicits prompt Th17 T responses to challenge with B. parapertussis and B. bronchiseptica.

**FIG 6:**
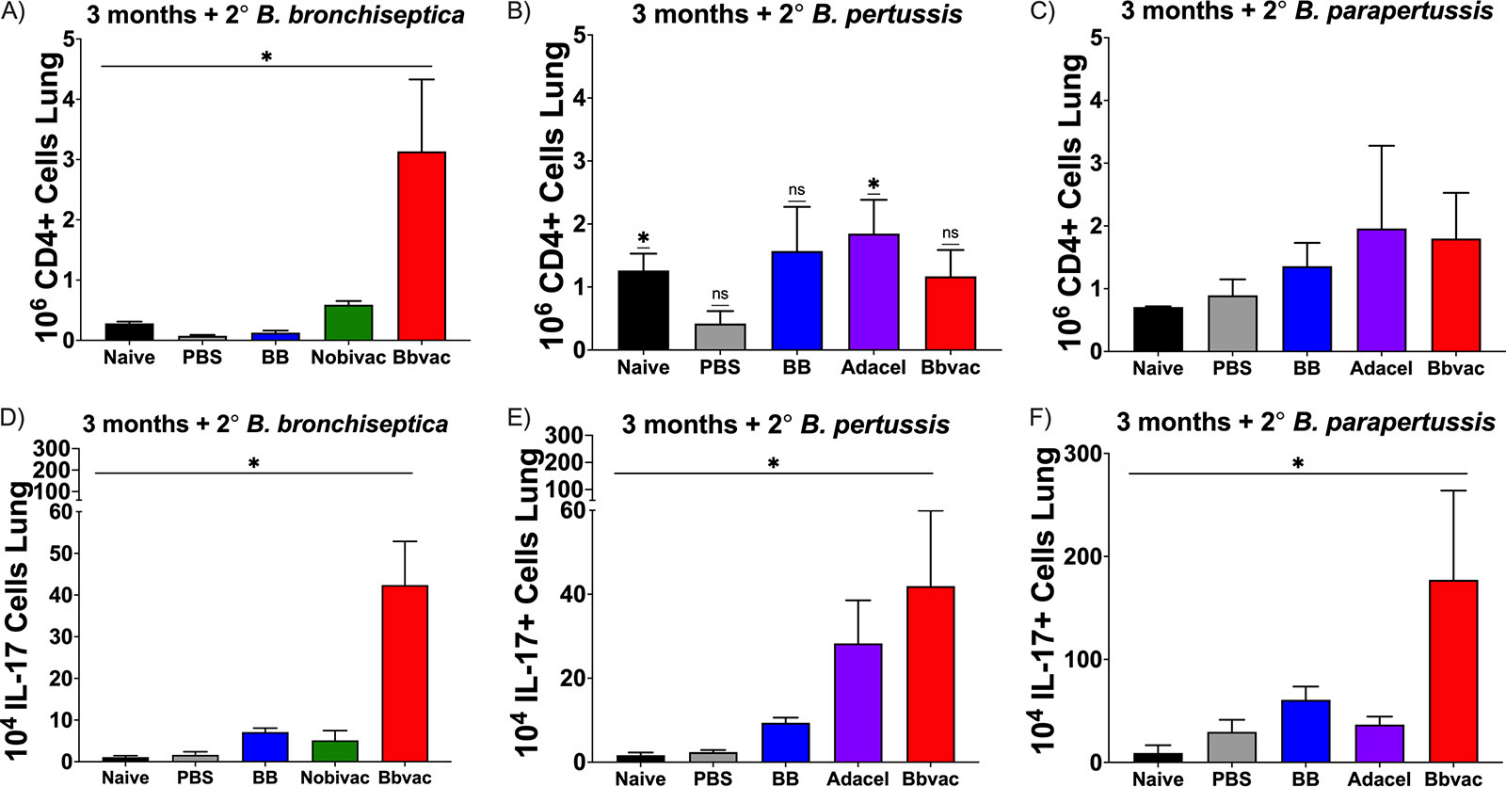
Bbvac promotes IL-17^+^CD4^+^ T cells. C57BL/6J were either vaccinated with PBS, vaccinated with the currently available vaccines Nobivac and Adacel, previously infected with B. bronchiseptica, or vaccinated with Bbvac. Three months postvaccination, mice were intranasally challenged with B. bronchiseptica (A and D), B. pertussis (B and E), or B. parapertussis (C and F). Mice were euthanized 7 days postchallenge to enumerate and characterize the CD4^+^ T cells in the lungs. Two-way ANOVA, Dunnett's multiple-comparison test. *, *P* < 0.05. Error bars represent standard error of the mean. *n* = 4 per strain and condition.

Adoptively transferred antibodies did not confer the full protection conveyed by Bbvac, consistent with the hypothesis that Th17-based cellular immunity is a critical component of the improved protection conferred by Bbvac (43, 49). To test this hypothesis, mice were vaccinated/challenged with Bbvac (red), B. bronchiseptica (blue), Adacel (purple), or Nobivac (green). Three months postvaccination, mice were challenged with B. bronchiseptica (Fig. 6A and D), B. pertussis (Fig. 6B and E), or B. parapertussis (Fig. 6C and F) as above. Seven days postinoculation, mice were euthanized and lungs dissected to compare the numbers of CD4-positive (CD4^+^) T cells and the fraction expressing interleukin 17 (IL-17).

Unvaccinated and unchallenged control mice (black) presented low basal numbers of CD4^+^ T cells in which the number of cells producing IL-17 was also low. As expected, sham vaccination (PBS, gray) did not increase the numbers of T cells in the lungs at day 7 postinoculation. However, vaccination with the currently available vaccines resulted in an increase in CD4^+^ T cells, especially when it followed a challenge with B. pertussis or B. parapertussis. The veterinary vaccine, Nobivac (green), led to a moderate increase of CD4^+^ T cells. Vaccination with Adacel (purple) resulted in a significant increase in the numbers of CD4^+^ T cells after challenge with B. pertussis (Fig. 6B) and B. parapertussis (Fig. 6C). Moreover, the number of those cells that produced IL-17 was significantly higher in response to B. pertussis (Fig. 6E) but not B. parapertussis (Fig. 6F). These results indicate that the animal vaccine Nobivac does not promote an increase in the number of CD4^+^ IL-17^+^ T cells, which can explain the need for repeated vaccinations to protect dogs.

Interestingly, previous infection with B. bronchiseptica (blue) did not increase the number of CD4^+^ T cells (Fig. 6A) producing IL-17 (Fig. 6D) in response to subsequent challenge with B. bronchiseptica; however, the numbers of CD4^+^ T cells increased in response to infection with B. pertussis when comparing with the sham vaccinated (Fig. 6B), with many of those being IL-17^+^ (Fig. 6E). Finally, vaccination with Bbvac (red) resulted in a significant increase in the number of CD4^+^ IL-17^+^ T cells as a response to B. bronchiseptica (Fig. 6A and D), B. pertussis (Fig. 6B and E) and especially B. parapertussis (Fig. 6C and F) challenge, respectively. These data indicate that Bbvac provides good levels of cellular immunity across the 3 species, increasing the numbers of CD4^+^ and CD4^+^ IL-17^+^ cells very rapidly following infection. It is noteworthy that despite the generally accepted view that convalescent immunity confers the best protection, our results show that the measurable outcomes of protection conferred by convalescence can be significantly improved upon.

### Extended protection conferred by Bbvac correlates with accumulation of interferon gamma-positive IL-17^+^ CD4^+^ T cells.

The above data indicate that Bbvac-induced protection involves a combination of humoral (Fig. 4 and 5) and cellular immunity (Fig. 6). Our previous data (Fig. 4) revealed that antibody titers remained relatively constant and are quickly recalled for at least 15 months postvaccination. To determine if the T cell IL-17 responses are also maintained over time, mice were vaccinated with Bbvac and, 15 months later, challenged with B. bronchiseptica, B. pertussis, or B. parapertussis. Mice were euthanized at day 7 postinoculation, and lungs were used to perform an intracellular staining to analyze the type of CD4 response produced in these mice.

**FIG 7:**
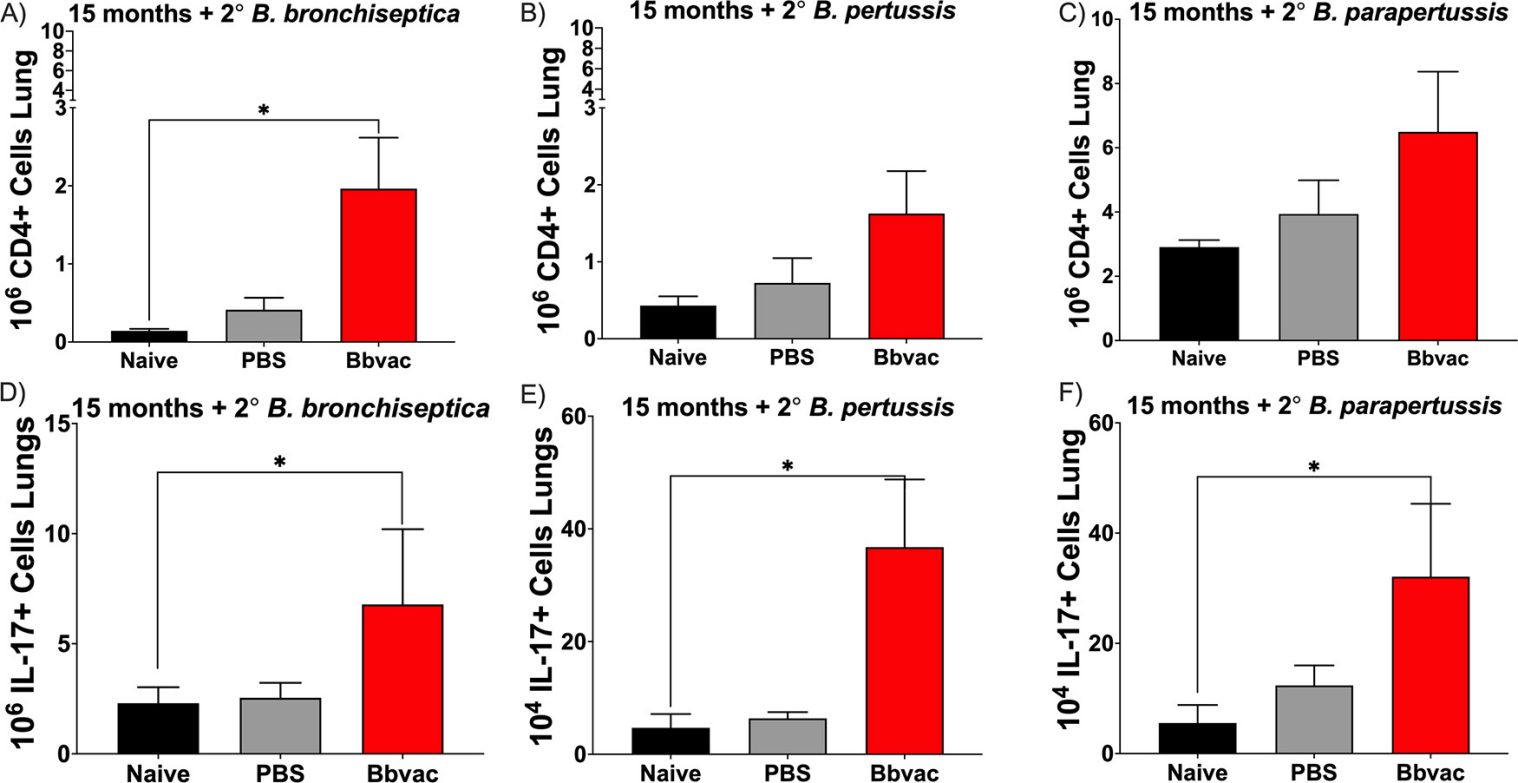
Bbvac promotes long-lasting IL-17^+^CD4^+^ T cell memory. C57BL/6J mice were vaccinated with PBS or vaccinated with Bbvac. Fifteen months postvaccination, mice were intranasally challenged with B. bronchiseptica (A and D), B. pertussis (B and E), or B. parapertussis (C and F). Mice were euthanized 7 days postinfection to enumerate and characterize the CD4^+^ T cells in the lungs. Two-way ANOVA, Dunnett's multiple-comparison test. *, *P* < 0.05. Error bars represent standard error of the mean. *n* = 4 per strain and condition.

As expected, uninfected control (black) and the sham PBS vaccinated control group (gray) showed no increase in numbers of CD4 nor CD4^+^ IL-17^+^ T cells 7 days following challenge with B. bronchiseptica (Fig. 7A and D), B. pertussis (Fig. 7B and E), or B. parapertussis (Fig. 7C and F). Remarkably, mice vaccinated with Bbvac exhibited significant increases in numbers of CD4^+^ IL-17^+^ T cells as a response to this brief 7-day infection. Interestingly, all the vaccinated mice challenged with either B. bronchiseptica (Fig. 7A and D), B. pertussis (Fig. 7B and E), or B. parapertussis (Fig. 7C and F) showed this increase, indicating that Bbvac induces a Th17 cellular mucosal response against the three classical *Bordetella* spp. that lasted for at least 15 months.

Overall, these results demonstrate that Bbvac confers better and more robust protection against the three classical *Bordetella* spp. The protection is due to a combination of cellular and humoral immunity that lasts at least 15 months. Importantly, this robust mucosal response protects against B. bronchiseptica, B. pertussis, and B. parapertussis, offering a unique vaccine that can provide cross-protection against all classical *Bordetella* spp.

## DISCUSSION

Convalescent immunity against B. pertussis in humans has been observed to provide robust and lasting protection against the pathogen. The first-generation reactogenic whole-cell vaccines (wP) comprised of inactivated bacteria formulations also provided protection that was reasonably effective and long-lasting. It has been established that the protective responses generated in response to vaccination with the wP vaccine correlate to a Th1/Th17 response pathway, inducing both cellular and antibody-mediated protection. Not surprisingly, efforts to improve the suboptimally performing acellular vaccines currently being used in several countries aim to emulate the Th1/Th17 pathway response of a natural infection, utilizing convalescent immunity and wP vaccines as the benchmarks in protection.

Protection conferred by the attenuated B. bronchiseptica live vaccine strain, Bbvac, is also based on the convalescent immunity of a self-resolving infection (3), but the measurable aspects of protection are significantly stronger than those derived from wild-type B. bronchiseptica. As our work here and earlier have shown, the immune response to Bbvac vaccination follows a Th1/Th17 pathway, which is robustly cellular and strongly antibody based and remains significantly responsive even 15 months following vaccination. As we have reported (2, 3, 39, 50), the mechanism of this enhanced host immune response is mediated via a loss-of-function deletion which renders Bbvac unable to modulate/suppress host immune responses like its wild-type counterpart. One major consequence of this pronounced host immune response is to render Bbvac a self-limiting infection (3), unlike its wild-type counterpart, which is a persistent colonizer of the upper respiratory tract in mammals. In this respect, intranasally delivered Bbvac is cleared from the nasal cavities of mice within 2 to 3 months, while the wild type is known to persist indefinitely in the nose.

Finally, the most consequential result of the enhanced immune response of Bbvac is the remarkable cross-species host protection it confers against all 3 of the classical *Bordetella* species. Currently deployed animal vaccines being used to protect against B. bronchiseptica provide limited and short-lived immunity, and Bbvac is already well positioned to undergo trials as a vaccine for domestic pets and animals. Yet its unique ability to confer protection against B. pertussis and B. parapertussis (and B. bronchiseptica) raises the exciting possibility that Bbvac itself, as a live vaccine, or knowledge gained from its study, could lead to a more robust vaccine against pertussis in humans. Unlike B. pertussis BPZE1 a promising live attenuated B. pertussis vaccine candidate currently being tested (20, 21), Bbvac is an attenuated derivative of the largely commensal B. bronchiseptica, posing even less risk to humans, and can protect against diverse *Bordetella* species. As the number of reported cases of respiratory diseases and other opportunistic infections caused by a growing list of zoonotic and environmental bordetellae is gradually increasing, it would be prudent to examine novel platforms to address this. The new strategy of Bbvac that focuses on disrupting bacterial immunosuppressive pathways to allow the host to induce broad, robust, and long-lasting protective immunity may be a step in this direction.

### Conclusions.

Whooping cough-like disease is unfortunately remerging (4, 5, 51) despite international vaccination efforts. In the manuscript, we describe a novel live attenuated vaccine, Bbvac, which profoundly outperforms the currently available vaccines, providing a combination of humoral and cellular immunity that lasts for at least 15 months in the murine model. Bbvac confers protection against at least the three classical *Bordetella* species, B. pertussis, B. parapertussis, and B. bronchiseptica, offering, for the first time, a vaccine candidate that confers cross-protection against several *Bordetella* spp. The findings demonstrate that by better understanding the molecular mechanisms that bacteria utilize to suppress the generation of adaptive protective immunity, we can develop alternative strategies for vaccine and therapeutic development.

## MATERIALS AND METHODS

### Bacterial strains and culture conditions.

Wild-type Bordetella bronchiseptica strain RB50 (B. bronchiseptica) and a derivative *btrS* mutant (RB50Δ*btrS*) (3), here called Bbvac (52), B. pertussis, and B. parapertussis were grown on plates of Difco Bordet-Gengou agar (BD; catalog no. 248200) supplemented with 20% defibrinated sheep blood from HemoStat and 20 μg/mL of streptomycin as previously described (1). For liquid cultures, B. pertussis and B. parapertussis were grown in Stainer-Scholte medium (1). B. bronchiseptica and Bbvac were grown in LB media (1). Details of the clinical strains included in this study are found in Table S2 in the supplemental material.

### Animal experiments.

We obtained 5- to 7-week-old wild-type C57BL/6J mice from Jackson Laboratories, Bar Harbor, Maine, or our breeding colony (established from Jackson Laboratories mice). Nasal cavity, trachea, lungs, spleen, and blood were collected postmortem. When tissues were used to enumerate colonies, collection was performed in beaded tubes, and following homogenization, colonies were enumerated in Bordet-Gengou agar supplemented with 20% defibrinated sheep blood from HemoStat and 20 μg/mL of streptomycin when plating laboratory strains; no streptomycin was used when plating clinical isolates. When organs were harvested for immunological studies, they were collected in 15-mL Falcon tubes containing sterile cell culture-grade PBS. Immune staining was performed as outlined below.

To evaluate the protection conferred by Bbvac, groups of mice were intraperitoneally vaccinated with PBS, one-fifth human dose of Adacel, or intranasal PBS containing 5 × 10^5^ Nobivac, Bbvac, or RB50. Ninety days postvaccination, mice were challenged with B. bronchiseptica, B. pertussis, or B. parapertussis. Mice were euthanized 7 days postchallenge to enumerate colonies and evaluate immune parameters. To evaluate convalescent immunity by rechallenge, nasal bacteria in primarily infected mice was eliminated by treatment with enrofloxacin (45 μg in 10 μL PBS) 2 doses daily for 5 days. Following this treatment, a control group was euthanized at day 80 to confirm clearance from the respiratory tract. The Bbvac-inoculated group was not treated (as clearance was observed by day 56), and a control group was euthanized at day 80 to confirm clearance. We collected nasal cavity, trachea, and lungs to enumerate CFU, and trachea was where we found higher dispersion due to the collection from the animals, which can render different amounts of tissue due to individual reasons.

### Enzyme-linked immunosorbent assays and cytokine assays.

Serum samples were utilized to evaluate antibody titers. We coated 96-well microtiter plates (Costar) with heat-killed B. bronchiseptica, B. pertussis, and B. parapertussis as previously reported (3). Serial dilution (2-fold dilutions) of the serum samples were added to the previously coated plate and were incubated at 37°. After several washes, IgG antibodies were purchased from Invitrogen. SureBlue (SeraCare; catalog no. 5120-0076) was added to start the developing reaction, which was terminated with HCl after 3 min. The plates were read at an optical density at 450 nm (OD_450_). The titer was determined to be the reciprocal of the lowest dilution in which an OD of 0.1 at 450 nm was obtained (3, 43, 49, 50).

### Flow cytometry.

Lungs were processed and stained as previously described (3, 44, 50). Numbers of live cells were enumerated with Countess II (Thermo Fisher) with trypan blue stain. Two million live cells were seeded in each well for staining (see Table S1). The acquisition of the data was performed using BD-LSR II (Becton, Dickinson), and analysis was done with FlowJo 10.0 following standard gating strategy (3, 44). Statistical significance was calculated using two-way analysis of variance (ANOVA) and Dunnett’s multiple-comparison tests in GraphPad Prism (3). Briefly, our gating strategy was single cells, live cells, T cells, CD4 versus CD8, intracellular CD4^+^ IL-17 interferon gamma (IFN-γ).

10.1128/msphere.00892-21.4 TABLE S1 List of antibodies used for flow cytometry experiments with the BD-LSR II (Becton Dickinson). This table includes target, clone, vendor, and catalog number. Download Table S1, DOCX file, 0.02 MB. Copyright © 2022 Gestal et al. 2022 Gestal et al. https://creativecommons.org/licenses/by/4.0/ This content is distributed under the terms of the Creative Commons Attribution 4.0 International license.

### Ethics statement.

This study was carried out in strict accordance with the recommendations in the Guide for the Care and Use of Laboratory Animals of the National Institutes of Health. Mice were bred and maintained at Paul D. Coverdell Center for Biomedical and Health Sciences, University of Georgia, Georgia (AUP, A2016 02-010-Y2-A3). All experiments were carried out in accordance with all institutional guidelines, and the protocol was approved by the Institutional Animal Care and Use Committee at the University of Georgia, Athens (*Bordetella*-host interactions AUPs, A2016 02-010-Y2-A6 and A2016 02-010-Y2-A3; breeding protocol, A2016 07-006-Y2-A5). All animals were anesthetized using 5% isoflurane and euthanized using carbon dioxide inhalation followed by cervical dislocation to minimize animal suffering. Animals were handled following institutional guidelines, in keeping with full accreditation from the Association for Assessment and Accreditation of Laboratory Animal Care International (3, 50).

### Statistical analysis.

All results were graphed in GraphPad Prism (version 9.2.0), and statistical significance was calculated using two-way ANOVA, Dunnett’s multiple-comparison tests; one-way ANOVA, Brown-Forsythe test; Bartlett’s test; or Mann-Whitney as indicated in each figure. For all our experiments, we used both genders (male and female) to account for gender variability. The number of animals used per experiment varied between experiments and is detailed in each figure legend.

### Data availability.

All data are available upon direct request.
