# Supplementary figures and images for: Bbvac: A Live Vaccine Candidate That Provides Long-Lasting Anamnestic and Th17-Mediated Immunity against the Three Classical Bordetella spp
Source: mSphere. 2022 Feb 23;7(1):e00892-21. doi: 10.1128/msphere.00892-21 (PMC8865921; doi:10.1128/msphere.00892-21)

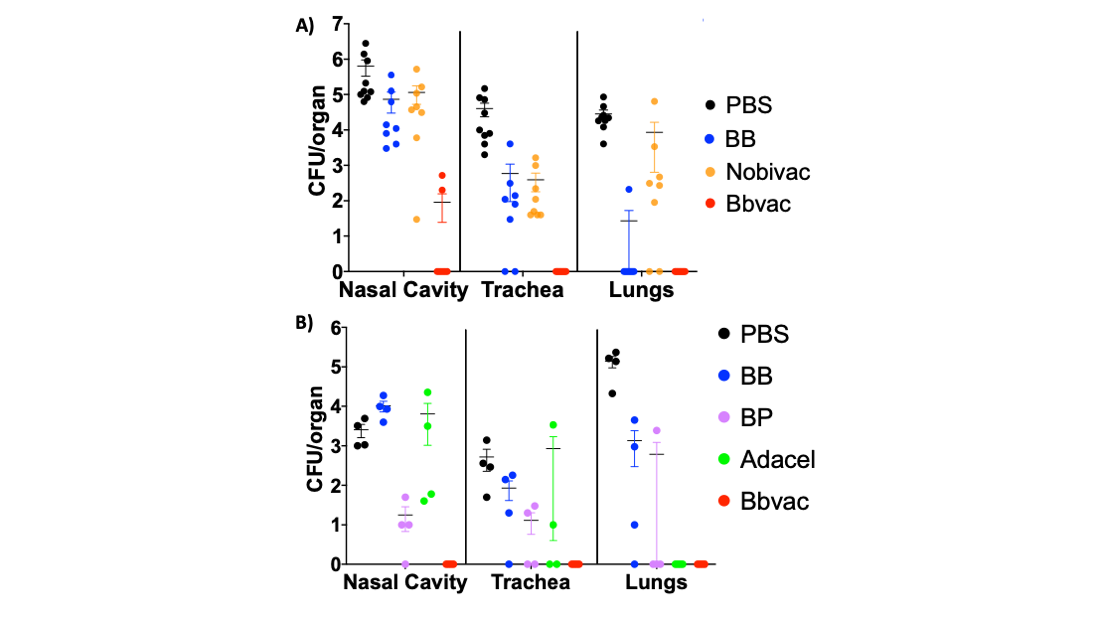

Supplement: FIG S1 [file msphere.00892-21-sf001.tif]

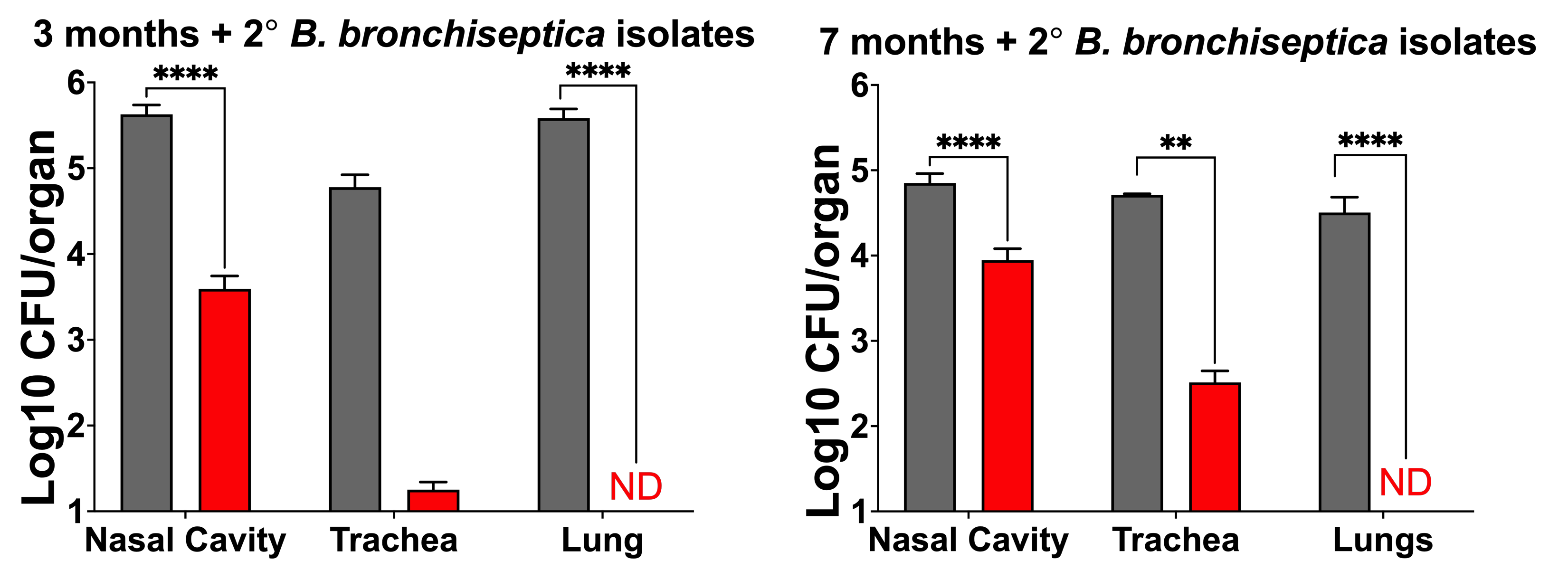

Supplement: FIG S2 [file msphere.00892-21-sf002.tif]

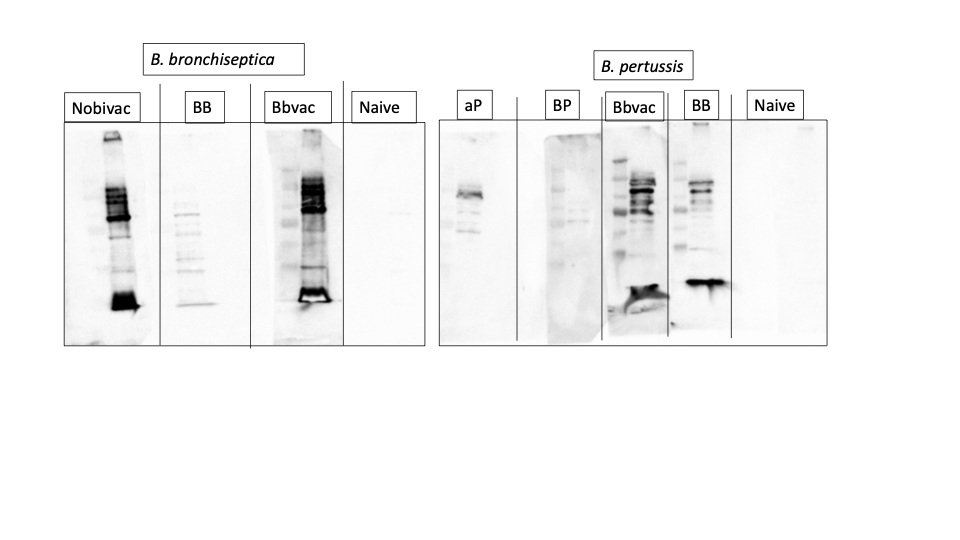

Supplement: FIG S3 [file msphere.00892-21-sf003.tif]
