# Supplementary material for: Bbvac: A Live Vaccine Candidate That Provides Long-Lasting Anamnestic and Th17-Mediated Immunity against the Three Classical Bordetella spp
Source: mSphere. 2022 Feb 23;7(1):e00892-21. doi: 10.1128/msphere.00892-21 (PMC8865921; doi:10.1128/msphere.00892-21)
Supplement: TABLE S2 [file msphere.00892-21-st002.docx]

**Table S2: Clinical isolates included in this study**. List of clinical Bordetella spp. strains utilize to determine protection levels conferred by Bbvac. These table indicate if the strains were used to test protection at 3 months, 7 months, or both.

| **Name of the isolate** | **Protection tested at** | **Reference** |
| --- | --- | --- |
| A309 | 3 & 7 | 48 |
| A310 | 3 & 7 | 48 |
| D443 | 3 & 7 | 48 |
| D444 | 3 | 48 |
| D447 | 3 | 48 |
| D448 | 3 | 48 |
| D449 | 3 | 48 |
| D495 | 3 | 48 |
| D755 | 3 & 7 | 48 |
| D758 | 3 & 7 | 48 |
| D759 | 3 & 7 | 48 |
| D760 | 7 | 48 |
| D762 | 7 | 48 |
| D973 | 7 | 48 |
| D974 | 7 | 48 |
| D980 | 7 | 48 |
| D982 | 7 | 48 |
