# Supplementary material for: Bbvac: A Live Vaccine Candidate That Provides Long-Lasting Anamnestic and Th17-Mediated Immunity against the Three Classical Bordetella spp
Source: mSphere. 2022 Feb 23;7(1):e00892-21. doi: 10.1128/msphere.00892-21 (PMC8865921; doi:10.1128/msphere.00892-21)
Supplement: TABLE S1 [file msphere.00892-21-st001.docx]

**Table S1: List of antibodies used for flow cytometry**. List of antibodies utilize to perform flow cytometry experiments with the BD-LSR II (Becton Dickinson). This table includes target, clone, vendor, and catalogue number.

| **Flow cytometry antibodies** | | | | |
| --- | --- | --- | --- | --- |
| **Fluorochrome** | **Target** | **Clone** | **Vendor** | **Reference** |
| PE | CD90.2 | 53-2.1 | BioLegend | 140308 |
| AF488 | CD4 | GK1.5 | BioLegend | 100423 |
| BV510 | CD8 | 53-6.7 | BD Biosciences | 563068 |
| FitC | IL-17 | TC11-18H10 | BD Biosciences | 560221 |
| PECy7 | INFγ | XMG1.2 | Tonbo | 60-7311 |
| PE | IL-10 | JES5-16E3 | BD Biosciences | 554467 |
| APC | IL-4 | 11B11 | Tonbo | 20-7041 |
